# Book Notices

**Published:** 1885-04

**Authors:** 


### BOOK NOTICES.

A SYSTEM OF MEDICINE, based upon the Law of Homœopathy. Edited by H. R. Arndt, M. D. Volume I, F. E. Boericke. Hahnemann Publishing House: Philadelphia, 1885.

Whatever opinions one may have upon the contested points of homœopathic practice, whether he be a loose prescriber or a strict Hahnemannian, he cannot fail to be proud of the handsome volume which Dr. Arndt here presents to his fellow practitioners.

It is a volume which, in extent and completeness, exceeds and excels anything of its kind yet published. Its descriptions of diseases are clear and full—amply full, although not so large as works like Ziemssen and Pepper's *System of Practical Medicine*, which are spun out to a tedious length.

This volume contains a General Introduction; a chapter on Physical Diagnosis (and a good chapter it is); also chapters on Diseases of the Respiratory Organs, the Diseases of the Organs of Circulation, and the Diseases of the Organs of Digestion. This last chapter includes the diseases of the mouth, teeth, gums, etc., the œsophagus, stomach, intestines, peritoneum, liver; most every abdominal organ has been treated of in this chapter except the poor spleen; he alone has been neglected.

While no intelligent physician will neglect the study of disease in all its myriad forms and phases, while admitting that no one should fail to study these branches of the medical art, we confidently claim that the homœopathic practitioner should be chiefly interested in the therapeutics of disease; and any work which is to be the practical assistant of the homœopathic practitioner should make clear and full the therapeutics of disease. This is a *most* difficult task—diseases vary so in their manifestations as they appear in various individuals. Pneumonia, as it appears in A, may require very different treatment from the same disease as manifested by B. This peculiarity of homœopathic practice—that any remedy be called for in any disease—can scarcely be better illustrated than by quoting from Dr. Erhman's *Cholera Epidemica*, where it is related that a desperate case of that desperate disease was cured by *Aconite*. The symptoms were "*complete suppression of pulse, voice. and urine, icy coldness of limbs*, general prostration, restlessness, with frightened and anxious looks." Few would even think of Aconite for such a condition; fever, heat being its leading indication to most careless prescribers. But, to return to our subject, in this volume the homœopathic treatment is very scantily considered; the indications are entirely too general.

On this subject the editor writes, in his Preface:

"Particular pains have been taken with the 'treatment' of the various diseases herein discussed. The remedies in most cases have been arranged, not alphabetically, as has been the custom, but in the order of their clinical importance. * * * *

"The indications for remedies are of necessity given with reference only to symptoms which occur in direct connection with the disorders treated; concomitant symptoms usually are ignored, because a work like this cannot be made to take the place of a work on symptomatology. In order to further increase the usefulness of this work, the chapters on 'Treatment' were not limited in range to therapeutics, but were made to embrace extensive observations on hygiene, nursing, dietetics, the use of hot and cold baths, electro-therapeutics, and the various means and agencies with which the intelligent medical man at this day combats disease and relieves suffering."

Perhaps nothing will give our readers a better idea of the varied therapeutics of this volume than will the list of contributors. Contributors to

Volume I are: Drs. H. R. Arndt, H. C. Clapp, Lucius D. Morse, J. S. Mitchell, A. K. Crawford, E. M. Hale, A. R. Thomas, W. T. Laird, C. M. Conant, A. C. Cowperthwaite, J. C. Gilchrist, E. U. Jones, W. H. Dickinson.

The best portion of the volume, to our mind, is the chapter on diseases of the organs of digestion. This is worth the price of the volume.

Dr. Arndt has facetiously classified homœopaths as of two kinds—the homœopaths and the Hahnemannians; this work will be more acceptable to the former than to the latter.

### PROCEEDINGS OF THE MINNESOTA STATE HOMŒOPATHIC INSTITUTE.

The proceedings of the Minnesota Institute, for the years 1883–84, have been collected into one volume. They include many interesting and useful papers.

